# Genomic evaluation methods to include intermediate correlated features such as high-throughput or omics phenotypes*

**DOI:** 10.3168/jdsc.2022-0276

**Published:** 2022-12-01

**Authors:** A. Legarra, O.F. Christensen

**Affiliations:** 1 GenPhySE (Genetique, Physiologie et Systemes d'Elevage), INRA, 31326 Castanet-Tolosan, France; 2 Center for Quantitative Genetics and Genomics, Aarhus University, 8830 Tjele, Denmark

## Abstract

Gene expression is supposed to be an intermediate between DNA and the phenotype, and it can be measured. Thus, for a trait, we may have intermediate measures, which are in fact a series of genetically controlled traits. Similarly, several traits may be measured or predicted using infrared spectra, accelerometers, and similar high-throughput measures that we will call “omics.” Although these measurements have errors, many of them are heritable, and they may be more accurate or easier to record than the trait of interest. It is therefore important to develop methods to use intermediate measurements in selection. Here, we present methods and perspectives for selection based on massively recorded intermediate traits (omics). Recent developments allow a hierarchical integrated framework for prediction, in which a trait is partially controlled by omics. In addition, the omics measures are themselves partly controlled by genetics (“mediated breeding values”) and partly by environment or residual factors. Thus, a part of the genetic determinism of a trait is mediated by omics, whereas the remaining part is not mediated, which results in “residual breeding values.” In such a framework, genetic evaluations consist of 2 nested genomic BLUP-based models. In the first, the effect of omics on the trait (which can be seen as an improved estimate of the phenotype) and the residual breeding values are estimated. The second model extracts the mediated breeding values from the improved estimate of the phenotype, considering that omics themselves are heritable. The whole procedure is called GOBLUP (genomics omics BLUP) and it allows measures in only some individuals; that is, it is a “single-step”-like method. In this model, heritability is split into “mediated” and “not mediated” parts. This decomposition allows us to predict how accurate the omics measure of the trait would be compared with the direct measure. The ideal omics measure is heritable and explains a large part of the phenotypic variation of the trait. Ideally, this could be the case for some traits with low heritability. However, even if the omics measure explains only a small part of the phenotypic variation, when omics measurement themselves are heritable, the use of such a model would lead to more accurate selection. Expressions for upper bounds of reliability given omics measurements are also presented. More studies are needed to confirm the usefulness of omics or high-throughput prediction. Usefulness of the technology likely needs to be checked on a case-by-case basis.

## Highlights

• There is now a plethora of biological “omics” and high-throughput new measurements.
• Total variability of the trait into omics-mediated and heritable components.
• From the theory, reliabilities can be derived for ideal cases.
• For selection purposes, it is better to have heritable omics than high explanatory ones.

## Graphical Abstract

**Figure:**
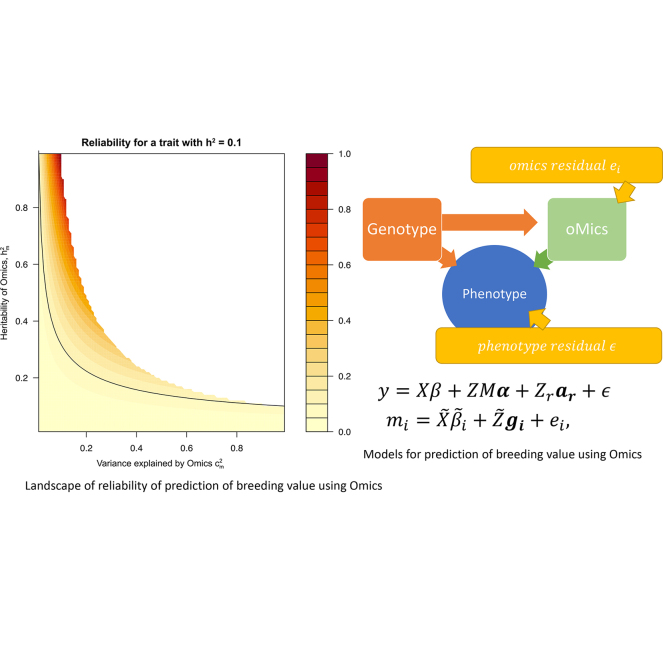
**Summary:** The effect of genes on phenotype is mediated by things such as RNA or metabolites. When part of these “omics” can be directly or indirectly measured, a proper modelling of this mediation can be written as 2 nested linear models, which split total variation and total heritability into mediated and not mediated fractions. Based on these partitions, it is possible to predict the accuracy of breeding value prediction using omics. This accuracy is a function on the part of variation explained by omics and on the heritability of the omics measurement.

Before the genomic selection era, collecting phenotypes was an arduous experience, and adding new traits to the breeding objective implied a cost-benefit consideration unless those traits were recorded for management purposes (Cole et al., 2021). The breeder had to live with “cheap” recordings (that required a fair amount of organization and coordination) and highly expensive (for the time being) computing procedures. Breeding objectives considered just a few traits (Cole et al., 2021).

Today, the situation is different for several reasons. First, breeding objectives are becoming more diverse (Cole et al., 2021) and they require more extensive phenotyping (Cole et al., 2021; Pérez-Enciso and Steibel, 2021). Second, the genomic revolution implied that high-throughput measurements could be dealt with by animal breeders through a mix of information flow (of genotypes), standardization (of DNA chips), computational power, and new or improved methods such as genomic (G)BLUP and single-step (ss)GBLUP (VanRaden, 2020). Finally, there is now a plethora of new measurements: some are closer to animal biology (e.g., gene transcripts, metagenome, images; Rutkoski et al., 2016; Morgante et al., 2020; Pérez-Enciso and Steibel, 2021) and some are less directly related to biology but can be easily obtained through sensor devices (e.g., spectra, accelerometers; O'Leary et al., 2020; Ricard et al., 2020; Bittante et al., 2022). In addition, recent developments (machine learning in particular) have opened the door to predict, in principle, almost anything from almost anything, which has prompted scholars to use more and more data. In the following, we use the word “omics” but we mean any complex set of measurements that could be seen as close to the biology of the trait of interest.

Assume then than we can do excellent work of predicting traits from a myriad of closer or indirect omics measures, whether these are gene transcripts, operational taxonomic unit counts in rumen or feces, accelerometer data, or milk spectra. How can these be converted into something usable for selection? A phenotype per se cannot directly be used to select animals. A fundamental principle in genetics (that, in our view, is sometimes disregarded) is that an animal transmits half its genotype to its offspring. This is the reason why natural and artificial selection act additively. Anything that is not contained in the DNA or cytoplasm of the female is not transmitted. For instance, the transcriptome may explain a large portion of a given phenotype such as growth. However, this transcriptome may be affected by environmental factors (e.g., food and management), which are not transmitted. Second, only a random gamete, half of the genotype, is transmitted—all dominant or epistatic combinations are lost, and many possible gametes exist. Thus, the breeding value (**BV**) or PTA is, literally, an expectation on random events (meiosis, mates, environments).

Hence, in addition to being able to predict phenotypes from omics, we also need a theory to use omics in genetic improvement of livestock. Recent developments (Weishaar et al., 2020; Christensen et al., 2021) led to prediction of BV (not of phenotypes) using intermediate data, and these developments also clarified relationships between heritabilities and the variance ratio explained by omics (e.g., “microbiabilities”). Before these publications, these relationships were not well understood. In addition to helping our understanding, a theory, even if not perfect, sets the stage for a priori plans for using omics in selection schemes from a few basic parameters.

In this work, we will (1) present a sketch of the theory and how it can be used for BV prediction, (2) discuss the circumstances in which the use of omics is advantageous with respect to current prediction based on phenotypes, (3) present some illustrative examples of omics use in plant and animal breeding, and (4) present some thoughts on selection schemes that use omics features. This review does not contain any studies with human or animal subjects and did not require animal care approval.

The development here is taken and condensed from Christensen et al. (2021), and we stick to its notation as much as possible. We use a linear model, which assumes that measurable observed covariates (belonging to a herd; temperature; omics; genotype at the marker, and so on) have measurable effects on a trait of interest. Whether these effects are “real” or “surrogates” of real effects (e.g., herd is a surrogate for farmer; SNP is a surrogate for QTL) is a question that we will not address here, where we will assume that effects are reasonably stable with respect to time (across a few generations) and space (from, say, Maryland farms to Georgia farms). This allows us to consider in the same framework “truly” biological effects (e.g., transcriptome) and surrogates of biology (e.g., infrared spectra).

A trait is classically decomposed as $y_{i}=a_{i}+e_{i}^{*},$ where *a* is an overall BV and $e_{i}^{*}$ is a residual (the part unexplained by genetics). Alternatively, if we knew all omics (**m**) that define the outcome of a trait (*y*), a basic model for individual *i* is $[y_{i}=m_{i}\alpha +\varepsilon_{i},$ where **m***_i_* contains (all relevant) omics measures for individual *i* and ***α*** contains their effects; we say that trait *y* is “mediated” by omics **m**. In addition, *ε_i_* is a residual, the part unexplained by omics. Note that *ε_i_* is different from $e_{i}^{*}$ as the 2 models are different. In any case, we cannot measure all relevant omics measures (e.g., some may happen during embryo development). Thus, we postulate a model in which the part unexplained by omics has some genetic determinism not mediated by omics, *a_r_* (where *r* indicates “residual”), leading to $y_{i}=a_{r\left(i\right)}+m_{i}\alpha +\varepsilon_{i}.$From this, we define a single omics value $u_{m\left(i\right)}=m_{i}\alpha$ (which is not a BV). In addition, omics measures are not transmitted to offspring; only genes controlling ***m*** are transmitted to offspring. Thus, omics (***m****_i_*) themselves need a decomposition into a genetic and a residual part, which leads to a another step in the hierarchy of models:

$$m_{i,j}=g_{i,j}+e_{i,j}.$$

The contribution of the BV *g_i,j_* of omics *j* to the phenotype is *g_i,j_α_j_*, whereas the contribution of the residual *e_i,j_* of omics *j* to the phenotype is *e_i,j_α_j_*. Thus, we can define an “omics-mediated” BV, *a_m(i)_*, as a sum over omics of *g_i,j_α_j_*: $a_{m\left(i\right)}=\sum_{j}\;g_{i,j}\alpha_{j}=g_{i}\alpha ,$which is, in fact, the genetic part of *u_m(i)_*. So, for each individual, there is a single omics-mediated value *u_m(i)_* and a “residual” BV *a_r(i)_* that explains the genetic variation of the phenotype part not mediated by omics; the same individual *i* has, for each omics *m_i,j_*, a BV *g_i,j_*; and the sum of the BVs for omics *g_i_* times their effects *α* gives the mediated BV $a_{m\left(i\right)};$ the overall BV is therefore $a_{i}=a_{r\left(i\right)}+a_{m\left(i\right)}.$

It is worth noting that assumptions of the model lead to uncorrelated *a_r_* and *a_m_*. This can be understood as follows. If gene A has action on the omics and the omics contribute to the trait, then gene A contributes to the genetic variation of *a_m_*, but not to that of *a_r_*. If gene B has no action on the omics yet it contributes to the trait (e.g., because the relevant pathway is not in the omics measurement), then gene B contributes to the genetic variation of *a_r_*, but not to that of *a_m_*. However, there is a correlation between each component *a_m_, a_r_*, and overall *a*, as shown later. Finally, the overall residual after discounting BV is $a_{i}=a_{r\left(i\right)}+a_{m\left(i\right)}.$ such that $y_{i}=a_{r\left(i\right)}+a_{m\left(i\right)}+e_{i}^{*}.$

The hierarchical model that we just presented is a generalization of models for genomic prediction: SNPs are omics measures with a heritability of 1. Alternatively, omics (**m**) can be seen as multiple traits, but instead of using massive multiple trait models with unstructured covariance matrices, we use a hierarchical model, which is actually a recursive model (a special case of simultaneous equation model; Gianola and Sorensen, 2004). The recursive model can be seen as a special, simplified case of multiple trait analyses, in which all covariances are described through regressions of one trait on another (Varona et al., 2007); in our case, these regressions are at the phenotypic level. Indeed, Saborío-Montero et al. (2020) used a recursive model to consider the relationship between metagenome and methane emission, but with only one measurement (relative abundance of a genera) at a time, with vague prior information on the regression coefficient. Instead of fitting one measurement at a time, Christensen et al. (2021) imposed a stricter prior information in which regression coefficient *α* values were drawn from a single distribution, as will be shown next. This allows simultaneous fitting and estimation of all omics measurements, and also an interpretation of associated variance components, as shown below.

Next, we need models to predict both **α** and **g**. First, we assume $Var\left(\alpha \right)=I\sigma_{\alpha }^{2}.$ It seems natural to assume that the effect of the transcript of one gene is a random effect. We also assume that the effect of the transcript of one gene is uncorrelated with that of another gene. However, assuming that the effect of a wavelength is different from that of a neighboring wavelength is more disputable. Second, we assume that omics measures are uncorrelated with each other; again, it is debatable whether this is reasonable or not and it needs to be verified with real data. Third, we assume constant heritability of omics (this assumption is easily removed at the cost of more complex algebra). The 3 assumptions lead to expressions for genetic evaluation that are quite easy to use and also interpretable in a quantitative genetics sense.

Christensen et al. (2021) presented a method for prediction (GOBLUP or Genomic Omics BLUP) based on 2 successive mixed model equations (**MME**). This is not an approximation, because the information from each MME is disjoined.

In the first step, omics effects on data are estimated, either by estimating omics effects (similar to SNP-BLUP) or using omics similarities (similar to GBLUP):

$$\left(\begin{array}{ccc} X^{\prime }X & X^{\prime }Z & X^{\prime }Z_{r}\\ Z^{\prime }X & {Z^{\prime }Z+G}_{M}^{-1}\xi_{1} & Z^{\prime }Z_{r}\\ {Z^{\prime }}_{r}Z & {Z^{\prime }}_{r}Z & {Z^{\prime }}_{r}Z_{r}+H^{-1}\xi_{2} \end{array}\right)\left(\begin{array}{c} \hat{\beta }\\ \hat{u}\\ {\hat{a}}_{r} \end{array}\right)=\left(\begin{array}{c} X^{\prime }y\\ Z^{\prime }y\\ {Z^{\prime }}_{r}y \end{array}\right);$$

$$\xi_{1}=\frac{1-c_{m}^{2}}{c_{m}^{2}};\;\xi_{2}=\frac{1-h_{r}^{2}}{h_{r}^{2}}.$$

For **X** and **Z** incidence matrices, $G_{M}$ is a scaled omics similarity matrix, $G_{M}=\frac{MM^{\prime }}{mean\left[diag\left(MM^{\prime }\right)\right]},$ and **H** is a genetic relationship (pedigree **A**, genomic **G**, or single-step **H**). Parameters are $c_{m}^{2}=\frac{\sum \sigma_{m}^{a}\sigma_{a}^{2}}{\sum \sigma_{m}^{a}\sigma_{a}^{2}+\sigma_{a,r}^{2}+\sigma_{ɛ}^{2}},$ the part of phenotypic variation explained by omics, and $h_{r}^{2}=\frac{\sigma_{a,r}^{2}}{\sum \sigma_{m}^{2}\sigma_{a}^{2}+\sigma_{a,r}^{2}+\sigma_{ɛ}^{2}},$ the part of phenotypic variation explained by “nonmediated” genetic effects; this model is not new (Guo et al., 2016; Difford et al., 2018). These equations yield the nonmediated part of the EBV $\left({\hat{a}}_{r}\right)$ and “improved phenotype predictions” $\left(\hat{u}\right),$ which are based on trait observations *y* and omics **M**, and can be seen as “*y* with less environmental noise,” or as a predictor trait such as SCS, which is a predictor of subclinical mastitis.

The notion of using a predictor of a trait instead of a direct measure is very old and is used, for example, for protein content (measured through milk spectra) or subclinical mastitis (measured through SCC). However, in contrast to these well-established uses, these phenotype predictions $\hat{u}$ may include animals with no phenotypes for *y* (which allows for early prediction of traits based on omics). Hayes et al. (2017), in fact, suggested calibrating prediction equations that used near infrared or nuclear magnetic resonance and then use the prediction as a correlated trait. However, this implies that predictions are portable through environments, years, and genetic backgrounds; the Christensen et al. (2021) proposal updates them continuously.

In the second step, once the phenotype predictors $\hat{u}$ are obtained, they are used as pseudo-traits in a second MME to extract the heritable part, ${\hat{a}}_{m}:$

$$\begin{array}{l} \left(\begin{array}{cc} X^{\prime }X & X^{\prime }\tilde{Z}\\ {\tilde{Z}}^{\prime }X & {\tilde{Z}}^{\prime }\tilde{Z}+H^{-1}\zeta \end{array}\right)\left(\begin{array}{c} \hat{\theta }\\ {\hat{a}}_{m} \end{array}\right)=\left(\begin{array}{c} {\tilde{X}}^{\prime }\hat{u}\\ {\tilde{Z}}^{\prime }\hat{u} \end{array}\right),\;\\ \zeta =\frac{1-h_{m}^{2}}{h_{m}^{2}}, \end{array}$$

with X and $\tilde{Z}$ being the design matrices for omics records, and parameter $h_{m}^{2}$ being the heritability of omics measurements. Total EBV is $\hat{a}={\hat{a}}_{m}+{\hat{a}}_{r}.$ The method has, in principle, been extended to single-step cases (not all animals are omics phenotyped), meaning that all cases are possible: animals with or without phenotypes, genotypes, or omics in all possible combinations. Extensions to more effects, multiple traits, and more complex covariance structures are immediate. Bayesian regressions such as Bayes B are also doable without much difficulty.

The whole procedure is called GOBLUP. Thus, the basic machinery for omics-based selection is there, even if omics features have not (yet?) been massively produced, with the possible exception of those in crop plants (Rincent et al., 2012; Guo et al., 2016; Robert et al., 2022). The next sections will explore the a priori usefulness of omics-based selection and illustrate some results from existing studies.

First, the linear model above with the simplifying assumptions explains the variance decomposition of 2 more popular models. First, GBLUP, with $y_{i}=a_{i}+e_{i}^{*},$ with $h^{2}=\frac{\sigma_{a}^{2}}{\sigma_{a}^{2}+\sigma_{e^{*}}^{2}},$ which is the classical analysis, and second, so-called GMBLUP or GTBLUP, where M stands for microbiome or metabolite and T for (gene) transcript (Guo et al., 2016; Difford et al., 2018) with $y_{i}=a_{r\left(i\right)}+m_{i}\alpha +\varepsilon_{i},$ which can equivalently be implemented using a “transcriptomic” similarity matrix of the form $MM^{\prime },$ from which $c_{m}^{2}=\frac{\sum \sigma_{m}^{2}\sigma_{a}^{2}}{\sum \sigma_{m}^{2}\sigma_{a}^{2}+\sigma_{a,r}^{2}+\sigma_{ɛ}^{2}},$ sometimes called microbiability (Difford et al., 2018) and $h_{r}^{2}=\frac{\sigma_{a,r}^{2}}{\sum \sigma_{m}^{2}\sigma_{a}^{2}+\sigma_{a,r}^{2}+\sigma_{ɛ}^{2}}$ (remaining heritability when omics are included). It has been empirically observed that moving from GBLUP to GTBLUP implied a drop in estimates of heritability (because omics are heritable) and a decrease in residual variance (Guo et al., 2016; Difford et al., 2018). Still, the relationship between this decrease and heritability of omics measurements was not well understood.

Christensen et al. (2021) showed that $h^{2}=c_{m}^{2}h_{m}^{2}+h_{r}^{2};$ in other words, omics capture $c_{m}^{2}$ of the total variability, which, times a fraction $h_{m}^{2}$ (heritability of omics measures), represents the genetic variation of the omics-mediated phenotype, whereas the nonmediated genetic part explains $h_{r}^{2}.$ In contrast, the ratio of residual variance to total variance reduces from $1-h^{2}$ to $1-h^{2}-\left(1-h_{m}^{2}\right)c_{m}^{2};$ in other words, conditional on omics, the trait is better explained. All of this has implications for selection that we will detail later.

Use of SNP chips for selection raises no questions in dairy cattle, but for species with a lower ratio of reproducer value to genotype cost, its use had to be considered. Similarly, we need to evaluate whether omics-based selection is useful given the cost of omics phenotyping and selection plans. In other words, is this a technology worth betting on?

The case for omics-based selection is similar to that for SNP-based selection. The breeder wants a measurement of the BV that is either more accurate or available earlier. Note that this is somehow different from plants or other uses (e.g., medical applications) where one is interested in the prediction of phenotype.

First, we want to know whether the omics-predicted phenotype is a good predictor of the actual phenotype; to give an example, can we predict phenotype of feed intake based on phenotypes of MIR spectra (Liu et al., 2022)? The squared correlation between the actual and omics-predicted trait is simply

$$r_{y,u}^{2}=\frac{Cov{\left(y,u\right)}^{2}}{Var\left(u\right)Var\left(y\right)}=\frac{Var\left(u\right)}{Var\left(y\right)}=c_{m}^{2},$$

the part explained by omics. To complete the preceding expressions, the squared genetic $\left(r_{a}^{2}\right)$ and residual $\left(r_{e}^{2}\right)$ correlations of the omics-predicted and the actual trait are derived. The squared genetic correlation is

$$r_{a}^{2}=\frac{h_{m}^{2}c_{m}^{2}}{h^{2}}=1-\frac{h_{r}^{2}}{h^{2}}.$$

In other words, when $h_{r}^{2}$ tends to 0, the genetic correlation tends to 1. Note that $r_{a}^{2}$ is (also) the squared correlation between the omics-mediated BV *a_m_* and the overall BV *a*, $r^{2}\left(a,a_{m}\right)$ $\left[andusingsimilararguments,r^{2}\left(a,a_{r}\right)=\frac{h_{r}^{2}}{h^{2}}\right].$ As for the squared residual correlation, this is

$$r_{e}^{2}=\frac{\left(1-h_{m}^{2}\right)c_{m}^{2}}{\left(1-c_{m}^{2}h_{m}^{2}-h_{r}^{2}\right)}=\frac{\left(1-h_{m}^{2}\right)c_{m}^{2}}{\left(1-h^{2}\right)}.$$

After an individual is phenotyped for omics, the omics measurements **m** are obtained. Plugging in estimates of omics effects $\hat{\alpha },$ a phenotypic prediction of $\hat{u}=m\hat{\alpha }$ is obtained. This is similar to indirect predictions on genomic selection based on markers. Then a prediction of BV can be obtained using *y*, $\hat{u},$ or both. In turn, this allows predictions for the trait of interest *y* and also BV prediction. We use this framework to characterize in which cases the omics feature is of interest using selection index theory. Assume that the unobserved omics trait *u* can be perfectly “predicted” conditionally on **m**; in other words, every *α_i_* is perfectly estimated. This will be the case, loosely speaking, when the product $c_{m}^{2}$ by the number of independent records is large; that is, the omics effect can be accurately estimated from records, and the trait of interest *y* has been recorded in a large number of individuals, and these individuals cover a large variation of the breed across herds, regions, and background genetics. In this case (*α* being perfectly estimated), phenotype prediction has reliability $r_{y,u}^{2}=c_{m}^{2}.$ This is already the case for traits that are very well predicted from milk spectra, such as fat content (Voort, 1980).

To get some perspective on reliability using omics data, we derived upper bounds of reliabilities considering simple examples of single animals. Ultimately, accuracies of bulls with daughters are a function of the number of daughters and the accuracies of these daughters; the same applies for marker estimates.

Cow Artxueta has a single record for *y*. Reliability of the EBV is simply $Rel_{y}=h^{2}=0.40.$ Heifer Bustintza has no record for *y* but has been properly phenotyped for omics, and *α* values are *exactly* known, so we have a perfect measure of *u*. The reliability of the phenotype prediction is $c_{m}^{2}.$ However, reliability of the EBV for *u* is actually the heritability of omics measurements $h_{m}^{2}.\;$ In turn, the reliability of the EBV for *y* is the reliability of the EBV for *u*, which is actually its heritability, $h_{m}^{2}=0.6,$ times the squared genetic correlation $r_{u}^{2}=\frac{c_{m}^{2}h_{m}^{2}}{h^{2}},$ resulting in $Rel_{m}={\left(h_{m}^{2}\right)}^{2}\frac{c_{m}^{2}}{h^{2}}.$

In this case, we can see that the space in which recording omics **m** is more reliable than measuring *y* is as follows: ${\left(h_{m}^{2}\right)}^{2}c_{m}^{2}>{\left(h^{2}\right)}^{2}.$ The breeder is therefore interested in using a set of omics measurements conceived such that all the genetic variation is mediated through omics $\left(h_{r}^{2}\Rightarrow 0\right),$ because, in that case, the ratio $\frac{c_{m}^{2}h_{m}^{2}}{h^{2}}=1-\frac{h_{r}^{2}}{h^{2}}$ tends to 1, and this increases accuracy based on omics measurements. Also, having heritable omics $\left(h_{m}^{2}\right)$ is more important than omics explaining a lot $\left(c_{m}^{2}\right),$ but again, we assumed that data sets were so large that *α* was correctly estimated anyway.

These ideas are reflected in Figure 1, which shows the reliability using omics (*Rel_m_*) for a low heritability (*h*^2^ = 0.10), in which case, *Rel_y_* = 0.10. The space in which omics are more accurate than the observation of the trait is wider when $h_{m}^{2}$ is high. This is exactly the case with genomic selection: SNPs have $h_{m}^{2}=1$ and $c_{m}^{2}=h^{2}$ when they explain all genetic variation of the trait.

**Figure 1:**
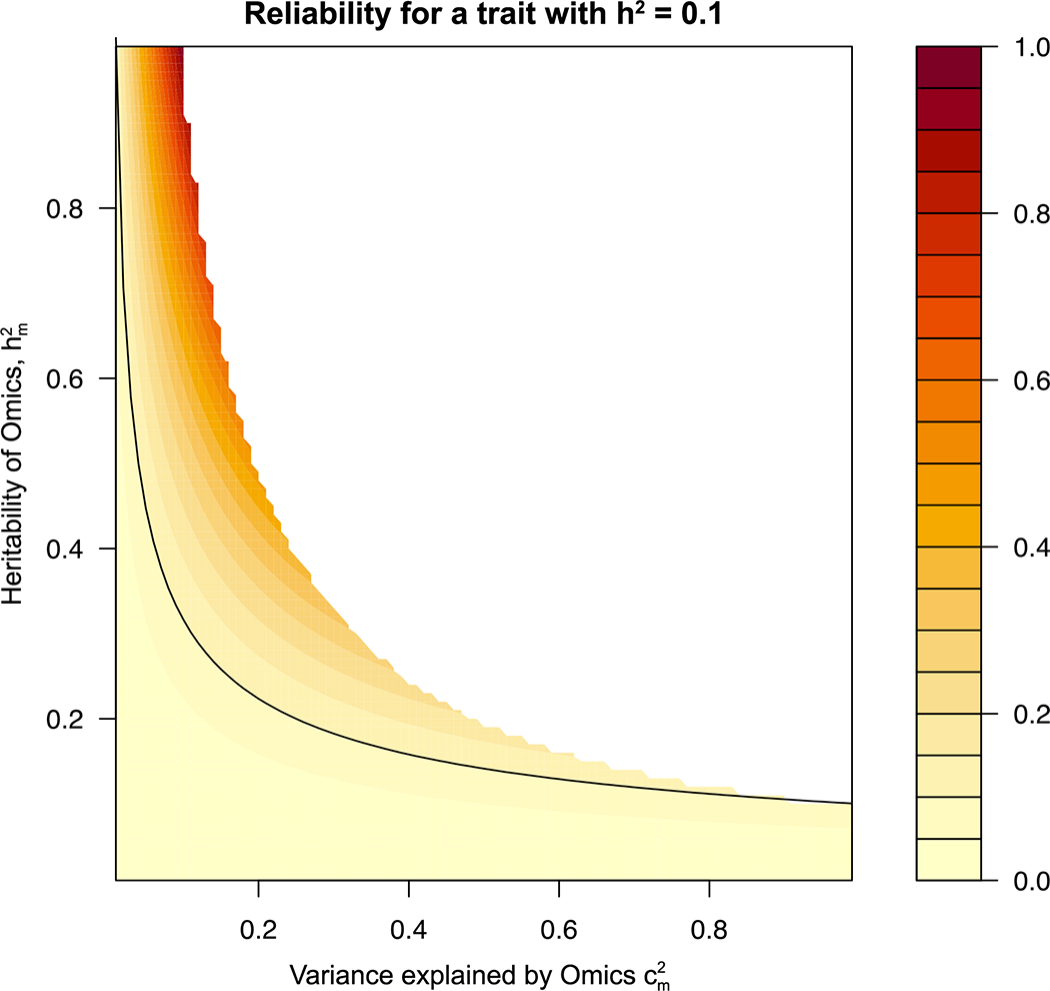
Landscape of reliability of prediction of breeding value using omics for a trait with *h*^2^ = 0.10 and changing values of $c_{m}^{2}$ and $h_{m}^{2},$ assuming that omics effects are estimated with no error. Points above the black line are those for which this prediction is more accurate than phenotype prediction.

Now consider cow Chinebral, which has both the record for *y* and the (perfect) prediction for *u*. According to selection index theory (Cameron, 1997), the reliability of a trait *Y* when traits *X* and *Y* are measured is as follows:

$$Rel\left(Y|X,Y\right)=\frac{h_{Y}^{2}+r_{A}^{2}h_{x}^{2}-2r_{P}r_{A}h_{X}h_{Y}}{\left(1-r_{P}^{2}\right)},$$

which in our context $\left[h_{Y}^{2}=h^{2};r_{A}^{2}=\frac{c_{m}^{2}h_{m}^{2}}{h^{2}};r_{P}^{2}=c_{m}^{2};h_{X}^{2}=h_{m}^{2}\right]$ results in

$$\begin{array}{c} Rel_{y,m}=\frac{h^{2}+\frac{c_{m}^{2}h_{m}^{2}}{h^{2}}h_{m}^{2}-2\sqrt{c_{m}^{2}\frac{c_{m}^{2}h_{m}^{2}}{h^{2}}h^{2}h_{m}^{2}}}{1-c_{m}^{2}}\\ =\frac{h^{2}+\left(\frac{h_{m}^{2}}{h^{2}}-2\right)c_{m}^{2}h_{m}^{2}}{1-c_{m}^{2}}. \end{array}$$

Now we provide some examples with actual and invented values. For instance, Guo et al. (2016) analyzed a trait (days to silking) with $h^{2}\approx 0.88,$ for which the heritability estimate dropped to $h_{r}^{2}\approx 0.385$ after fitting transcriptome measurements, which were highly explanatory $\left(c_{m}^{2}\approx 0.55\right),$ and were themselves quite heritable $\left(h_{m}^{2}\approx 0.90\right).$ In a study in mice, Perez et al. (2022) report for the trait BW10, $h^{2}=0.42,$ whereas $c_{m}^{2}=0.54$ and $h_{m}^{2}=0.50,$ from which we deduced $h_{r}^{2}=0.15.$

Then we considered the case of a low-heritable trait $\left(h^{2}=0.05\right)$ for which there are 2 options. An omics measure of low heritability $\left(h_{m}^{2}=0.10\right)$ explains a good portion of the phenotypic variation $\left(c_{m}^{2}=0.50\right).$ An alternative omics measure of high heritability $\left(h_{m}^{2}=0.50\right)$ explains a small portion of the phenotypic variation $\left(c_{m}^{2}=0.10\right).$

With these elements (presented in Table 1) and assuming that omics effects can be perfectly estimated, we can estimate the reliabilities using either an animal's own phenotype, omics data, or both (Table 2). For the real-data cases in mice and maize, using the omics record is not more accurate for EBV estimation than the phenotypic record, which is itself rather heritable. However, the EBV omics prediction is quite reliable and could be used if it were less expensive or could be measured earlier in life (which is often the case in crops). When variance components resemble the mice case, our results show that combining information from the actual phenotype and record would yield more accurate predictions.

**Table 1:** Scenarios with different variance components for phenotype and breeding value prediction^1^

Variance component	Maize^2^	Mice^2^	Low $h^{2},$ high $c_{m}^{2},$ low $h_{m}^{2}$	Low $h^{2},$ low $c_{m}^{2},$ high $h_{m}^{2}$
$h^{2}$	0.88	0.42	0.05	0.05
$h_{m}^{2}$	0.90	0.50	0.10	0.50
$c_{m}^{2}$	0.55	0.54	0.50	0.10
$h_{r}^{2}$	0.385	0.15	0	0

1 $h^{2}$ = heritability of the trait; $c_{m}^{2}$ = variance explained by omics; $h_{m}^{2}$ = heritability of omics; $h_{r}^{2}$ = heritability of the trait not mediated through omics.

2 Maize parameters are from Guo et al. (2016) and mice parameters from Perez et al. (2022)

**Table 2:** Reliabilities of phenotype and breeding value prediction in 4 cases with parameters detailed in Table 1^1^

Case	Maize	Mice	Low $h^{2},$ high $c_{m}^{2},$ low $h_{m}^{2}$	Low $h^{2},$ low $c_{m}^{2},$ high $h_{m}^{2}$
Phenotype prediction, own record	0.88	0.42	0.05	0.05
Phenotype prediction, omics	0.55	0.54	0.50	0.10
Breeding value prediction, own record	0.88	0.42	0.05	0.05
Breeding value prediction, omics	0.51	0.32	0.10	0.50
Breeding value prediction, own record + omics	0.88	0.44	0.10	0.50

1 $h^{2}$ = heritability of the trait; $c_{m}^{2}$ = variance explained by omics; $h_{m}^{2}$ = heritability of omics; $h_{r}^{2}$ = heritability of the trait not mediated through omics.

The invented trait gives more insights. The omics with high $c_{m}^{2}$ is quite reliable for phenotype prediction but not as reliable for BV prediction. In the case where omics explain less of the trait but are more heritable, the phenotype prediction is not particularly good but the BV prediction is quite accurate. (A caveat here is that this is somehow misleading, because in practice the accuracy of estimation of omics effects *α*, which we assumed to be perfect, depends on $c_{m}^{2}).$ In any case, Table 2 illustrates that for selection purposes, it is more important to have heritable omics measures than explicative ones.

Finally, there is abundant literature related to phenotype prediction (Guo et al., 2016; Lane et al., 2020; Perez et al., 2022) but the genetic interpretation of the phenotype prediction in that literature is very scarce. In crop breeding (Guo et al., 2016; Hayes et al., 2017; Rincent et al., 2018), obtaining biochemical measures from grains is easy. However, studies focus mainly on phenotypic prediction because, on the one hand, crop breeders tend to analyze single-generation experiments (unlike dairy cattle breeders) and, on the other hand, field trials are expensive and complicated to set up, so a phenotypic prediction is very useful. The literature in livestock genetics is less abundant because the only cheap available data are milk spectra (Liu et al., 2022). However, hard-to-measure traits have been modeled through closer biological measures such as metagenomic measures (Difford et al., 2018; Buitenhuis et al., 2019).

Another interesting use of prediction with intermediate features is to select differently for the mediated and not-mediated components of the trait. For instance, Weishaar et al. (2020) suggested, in a microbiota context, that selecting mediated BV (*a_m_*) will change microbiota composition (which may compromise rumen health), whereas selecting residual BV (*a_r_*) “will likely improve the trait by improved metabolic efficiency” (which may compromise overall health). These aspects could be taken into account for the construction of selection indices.

Overall, using omics or high-throughput measures may not be a “one size fits all” method but we consider it worth further exploration. The theory presented in this paper for BV prediction and the theory sketched for reliability of such predictions can help researchers determine when using omics or high-throughput measures is worthwhile for selection.
